# Sex differences in microvascular patterns associated with low- and high-risk for cardiovascular disease

**DOI:** 10.1186/s13293-026-00955-0

**Published:** 2026-07-27

**Authors:** Elin Nyman, Rasmus Magnusson, Tomas Strömberg, Carl Johan Östgren, Sara Bergstrand, Hanna Jonasson

**Affiliations:** 1 Department of Biomedical Engineering, Linköping University, Linköping, Sweden; 2 Department of Health, Medicine and Caring Sciences, Linköping University, Linköping, Sweden; 3 Centre of Medical Image Science and Visualization (CMIV), Linköping University, Linköping, Sweden

**Keywords:** Microcirculation, Sex differences, Risk factors, Cardiovascular disease, Post-occlusive reactive hyperemia, Skin laser Doppler flowmetry

## Abstract

**Background:**

The role of the microcirculation in cardiovascular health in males and females is not fully understood. Skin microvascular function can be assessed non-invasively by measuring the response to a brief occlusion of the arm, which produces a hyperemic response that depends on multiple regulatory pathways. Whether the resulting time-resolved microvascular parameters discriminate between low- and high-cardiovascular-risk individuals, and whether the informative features differ between males and females, is not known.

**Method:**

We analyzed time-resolved skin microcirculatory hemoglobin oxygen saturation and perfusion measured using a combined system integrating laser Doppler flowmetry and diffuse reflectance spectroscopy during a standardized occlusion-release protocol (including baseline, occlusion and reperfusion phases with a total duration of 20 min) in 3,177 participants in the Swedish CArdioPulmonary bioImage Study (SCAPIS) microvascular sub-study. Participants were stratified into a low-risk group (Systematic COronary Risk Evaluation 2 (SCORE2) < 5%, no diabetes or previous atherosclerotic cardiovascular disease (ASCVD); *n* = 1,645) and a high-risk group (SCORE2 > 7.5%, or diabetes, or previous ASCVD, or severe chronic kidney disease; *n* = 761). Random forest classifiers were trained to discriminate low- from high-risk individuals using the full time-resolved signals, in the combined cohort and separately by sex, with feature importance mapped back to the time axis.

**Results:**

The classifier achieved area under the curve (AUC) of 0.685 (95% confidence interval (CI): 0.663–0.707) in the full cohort (i.e. including both females and males). Discrimination was primarily driven by the post-occlusion hyperemic phase of oxygen saturation, with lower responses observed in high-risk individuals compared to low-risk individuals. Sex-stratified models achieved similar performance in females (AUC 0.638, 95% CI: 0.596–0.679) and males (AUC 0.644, 95% CI: 0.628–0.660). In males, both perfusion and oxygen saturation contributed to risk discrimination, with low-risk individuals exhibiting a more pronounced hyperemic perfusion response. In females, discrimination was driven mainly by oxygen saturation, while perfusion responses showed substantial overlap between risk groups and limited discriminatory value.

**Conclusions:**

Time-resolved microvascular signals provide information for cardiovascular risk discrimination that differs between males and females. In both sexes, high-risk individuals exhibited an attenuated post-occlusion oxygen saturation response, indicating impaired microvascular reactivity. Perfusion contributed additional discriminatory information in males, but showed limited value in females. These findings suggest that perfusion and oxygen saturation reflect partly different aspects of microvascular regulation, with different relevance to cardiovascular risk in males and females.

## Abstract (plain language summary)

The smallest blood vessels in the body, called the microcirculation, deliver oxygen to tissues and respond to changes in demand. How well the microcirculation works is linked to long-term cardiovascular health, and growing evidence suggests it functions differently in females and males. We studied skin microvascular function in over 3,000 middle-aged adults from a Swedish population study. Using a blood pressure cuff on the upper arm, we briefly stopped the blood flow and then measured how oxygen levels and blood flow recovered in the skin afterwards. We compared people at low and high risk of future cardiovascular disease, separately for females and males, using a machine learning method called a random forest. The machine learning models could distinguish low- from high-risk individuals based on these microvascular signals. Importantly, the most informative parts of the signal were different in females and males: oxygen levels carried useful information in both sexes, but blood flow recovery only mattered for males. These findings suggest that the microcirculation responds in sex-specific ways and that this should be taken into account in cardiovascular research.

## Highlights

- Time-resolved skin microvascular parameters during post-occlusive reactive hyperemia discriminated low- from high-cardiovascular-risk individuals in 3,177 participants from the SCAPIS cohort.
- Oxygen saturation in the post-occlusion hyperemic phase was the dominant informative feature for risk classification in the combined cohort.
- Sex-stratified random forest models achieved similar performance in females and males, but relied on different microvascular parameters.
- Oxygen saturation contributed to risk discrimination in both sexes, whereas perfusion contributed only in males.

## Introduction

Cardiovascular disease (CVD) remains the leading cause of morbidity and mortality worldwide, but its incidence, presentation, and prognosis differ substantially between males and females. Across adulthood, males typically exhibit a higher burden of atherosclerotic disease until midlife, after which females’ risk accelerates and eventually surpasses that of males following the menopause transition [1, 2].

These epidemiologic patterns have traditionally been framed in terms of differences in obstructive epicardial coronary artery disease and large artery atherosclerosis. However, a growing body of work indicates that microcirculation plays a central role, with sex-specific differences shaping not only local tissue perfusion but also systemic hemodynamics, cardiac remodeling, and clinical phenotypes [3] such as ischemic heart disease [4], heart failure with preserved ejection fraction [5], and cardiometabolic complications [6]. The microcirculation is the principal site of vascular resistance and of dynamic matching of blood flow to tissue metabolic demand. Its structure, function, and regulatory mechanisms are all modulated by sex hormones and differ between the sexes [1–4].

The updated Systematic COronary Risk Evaluation 2 (SCORE2) is a risk prediction model that estimates the 10-year risk of fatal and non-fatal CVD in adults aged 40–69 without prior CVD or diabetes [7]. It was developed separately for males and females and uses age, sex, smoking status, systolic blood pressure, and non-HDL cholesterol. SCORE2 is widely used in primary care to identify individuals at risk and guide preventive strategies. Stratifying study participants by SCORE2 into low- and high-risk groups has recently been used to investigate whether biomarker patterns associated with subclinical atherosclerosis differ between risk profiles [8], an approach that can reveal mechanisms not captured by the risk score itself.

We have previously shown sex-specific differences in post-ischemic skin peak hemoglobin oxygen saturation (OxyP) [9] and that OxyP is associated with cardiovascular risk factors [10]. In individuals without diabetes and previous CVD, those in the lowest OxyP quartile (Q1) had a fivefold higher risk of having a SCORE2 > 10% compared to those in the highest quartile (Q4) (odds ratio (OR): 4.96, 95% confidence interval (CI): 2.76–8.93) when adjusting for confounders, as well as to a higher extent having a coronary artery calcium score (CACS) > 0 [11].

Microcirculatory responses to stimuli such as post-occlusive reactive hyperemia (PORH) are inherently dynamic: the shape of the reperfusion curve, the timing and magnitude of the hyperemic overshoot, and the return to baseline all carry information about vascular function that a single scalar summary (peak, mean, area) cannot fully capture. In other cardiovascular domains, analyzing the full time-resolved signal rather than extracted scalar features has revealed disease information that manual parameter extraction missed, for example in continuous intensive care unit (ICU) vital-sign monitoring [12]. Machine learning methods, including random forests and neural networks, are well suited to this kind of data and have been applied to a range of cardiovascular risk prediction tasks [8, 13], but to our knowledge they have not been used to analyze stimulus-response recordings of microcirculatory perfusion and oxygen saturation in relation to CVD risk.

In this study, we investigated whether time-resolved microvascular signals recorded during PORH can discriminate between low- and high-risk CVD groups defined by SCORE2 in a large population-based cohort, and whether the informative features differ between males and females.

## Materials and methods

### Study population

This study utilized data from the Swedish CArdioPulmonary bioImage Study (SCAPIS). SCAPIS is a nationwide, population-based, cross-sectional cohort designed to improve risk prediction and increase understanding of mechanisms underlying cardiopulmonary disease, comprising 30,154 individuals aged 50–64 years, randomly selected from the general population register between 2013 and 2018. Recruitment was conducted across six university hospital regions in Sweden (Göteborg, Linköping, Malmö/Lund, Stockholm, Umeå, and Uppsala). Participants underwent extensive and standardized baseline examinations, including imaging of the cardiovascular and pulmonary systems, physiological measurements, and detailed questionnaires on lifestyle and health. The overall study design and baseline assessments have been described in detail previously [14]. SCAPIS was approved by the Regional Ethics Committee at Umeå University (Dnr 2010-228-31 M with amendment, EPN Umeå) and conducted in accordance with the Declaration of Helsinki.

A local add-on study to SCAPIS, focusing on microcirculatory assessment, was conducted at Linköping University Hospital between January 2016 and June 2018. This sub-study included 3,809 participants residing in the municipality of Linköping. Ethical approval was obtained from the Regional Ethics Committee in Linköping (Dnr 2018/156 − 31) and all participants provided written informed consent prior to participation.

### Assessment of cardiovascular risk factors

Cardiovascular risk factors were assessed using both self-reported data and standardized measurements obtained during SCAPIS examinations. Self-reported data such as personal health history, current medication, lifestyle habits and smoking status were collected through a questionnaire. These data were used to identify participants with previously diagnosed dyslipidaemia, hypertension, and/or diabetes. An overnight fasting venous blood sample was collected and analyzed for plasma glucose, HbA1c, total cholesterol, high-density lipoprotein (HDL) cholesterol, triglycerides, creatinine and high-sensitivity C-reactive protein (hsCRP). Participants without a prior diabetes diagnosis but with plasma glucose ≥ 7.0 mmol/L and/or HbA1c ≥ 48 mmol/mol were classified as having newly detected diabetes.

### Microvascular assessment

Microvascular function was assessed using a PORH protocol. Participants were instructed to refrain from large meals and coffee for at least three hours, nicotine for four hours, and alcohol for twelve hours prior to the examination. They were also asked to omit medication on the study day except for anticoagulants, contraceptives, or drugs prescribed for chronic pain, diabetes, epilepsy, Parkinson’s disease, or spasticity. Upon arrival, participants rested in a supine position for at least fifteen minutes before the start of measurement. The examination room temperature was maintained at 23.7 ± 0.5 °C.

The protocol included 5 min of baseline recording, 5 min of brachial arterial occlusion at 250 mmHg, and 10 min of reperfusion. Skin microcirculation was continuously recorded from the volar forearm using a PeriFlux 6000 EPOS system (Perimed AB, Järfälla, Sweden) and a fiber-optic probe (Perimed AB, Järfälla, Sweden), positioned to avoid moles, hair, and veins and held in place by double-adhesive tape. The PeriFlux 6000 EPOS system (Perimed AB, Järfälla, Sweden) integrates laser Doppler flowmetry (LDF) and diffuse reflectance spectroscopy to quantify hemoglobin oxygen saturation, red blood cell (RBC) tissue fraction, and microvascular perfusion in real time. Using a model-based approach that fits measured diffuse reflectance and laser Doppler spectra to multi-parameter skin model, these parameters can be expressed in absolute units, in contrast to conventional LDF which provides measurements in arbitrary units [15, 16]. The reported perfusion reflects total microvascular perfusion, i.e. the concentration of moving red blood cells times their average velocity. PORH peak values for oxygen saturation (OxyP) and perfusion were defined as the highest value reached after release of the cuff [9].

Detailed exclusion criteria for the microcirculation measurement have been described previously [10, 11]. Measurements were excluded if the protocol was aborted, if technical errors occurred (poor model fit, uncertain RBC tissue fraction, data acquisition failure, missing files), if protocol deviations were detected (motion artefacts, incomplete occlusion, incorrect occlusion time), or if participants did not follow pre-examination instructions. After these exclusions, 3,300 individuals remained. An additional 123 participants were excluded due to incomplete time-series data (missing values or insufficient recording duration), leaving 3,177 participants for analysis.

### Risk stratification

Cardiovascular risk was assessed using the SCORE2 algorithm, which estimates the 10-year risk of fatal and non-fatal CVD and is calibrated to country-specific risk regions. Sweden is classified as a moderate-risk region. In individuals aged 50–69 years, SCORE2 < 5% is considered low-to-moderate risk, 5% to < 10% high risk, and ≥ 10% very high risk [17].

To obtain two clearly separated risk strata and ensure adequate sample size in subgroup analyses, we used slightly stricter cutoffs than the conventional categories. A low-risk group was defined as participants with SCORE2 < 5%, without diabetes mellitus (DM) and without previous atherosclerotic cardiovascular disease (ASCVD) (myocardial infarction, stroke, coronary artery bypass grafting or percutaneous coronary intervention). A high-risk group was defined as participants with SCORE2 > 7.5%, or with DM, or with previous ASCVD, or with severe chronic kidney disease (eGFR < 30 mL/min/1.73 m²). The lower high-risk threshold (7.5% vs. 10%) was chosen to ensure sufficient sample size in the high-risk female subgroup, consistent with previous work [8].

Participants with SCORE2 between 5% and 7.5%, without additional risk-defining conditions fell between the two strata and were not included in the analysis.

Of the 3,177 participants with valid microvascular measurements, 1,645 met the low-risk criteria and 761 met the high-risk criteria.

### Data handling

The time-resolved hemoglobin oxygen saturation and speed-resolved perfusion during the PORH protocol were used as input to the classification models, with the two risk groups defined above (low-risk or high-risk), as the target label. Each signal was resampled to 2 Hz, resulting in 2,380 data points per participant, which were used as input features for the model.

**Fig. 1:**
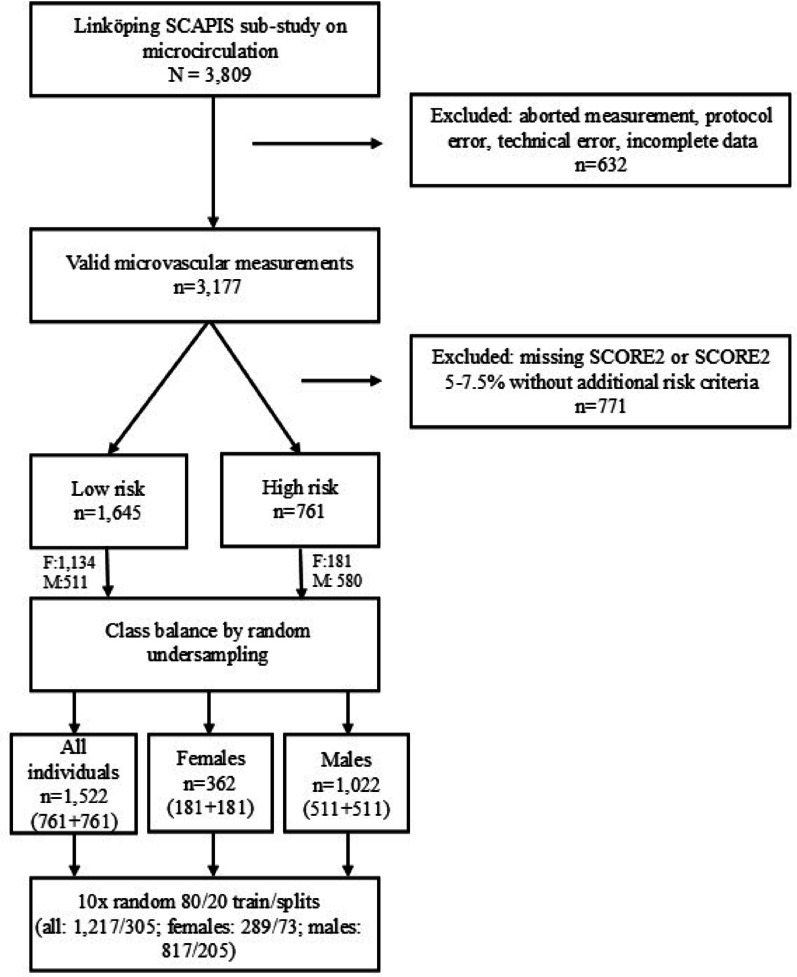
Flow diagram of participant inclusion and data handling. Of 3,809 participants in the Linköping SCAPIS microvascular sub-study, 3,177 had valid time-resolved oxygen saturation and perfusion measurements after quality checks. Participants were stratified into a low-risk group (SCORE2 < 5%, no diabetes or previous ASCVD; *n* = 1,645) and a high-risk group (SCORE2 > 7.5%, or diabetes, or previous ASCVD, or severe chronic kidney disease; *n* = 761). Participants missing SCORE2 data or with a SCORE2 between 5% and 7.5% without additional risk-defining conditions were not included. Three analysis datasets were constructed (all individuals, females only, males only) and balanced by random undersampling of the majority class. Each balanced dataset was split 80/20 into training and test sets, with the undersampling and split repeated independently 10 times. F - females, M - males

Analyses were performed on three datasets: all individuals (low risk *n* = 1,645, high risk *n* = 761), females only (low risk *n* = 1,134, high risk *n* = 181), and males only (low risk *n* = 511, high risk *n* = 580) (Fig. 1). Because the classes were unbalanced, we balanced the classes by randomly undersampling the majority class to match the minority class size. This yielded balanced datasets of *n* = 1,522 (all), *n* = 362 (females), and *n* = 1022 (males). To account for variability due to data partitioning and undersampling, we repeated the undersampling and 80/20 train/test split independently 10 times for each dataset (all: 1,217/305; females: 289/73; males: 817/205). All model performance metrics and feature importance were averaged across the 10 splits. 95% confidence intervals were calculated from the standard deviation across splits using the t-distribution (df = 9).

### Machine learning models

Random forest classifier models (scikit-learn 1.8.0, RandomForestClassifier) were trained to discriminate low- from high-risk individuals using the time-resolved oxygen saturation and perfusion signals as input. Each forest consisted of 300 decision trees to ensure stability and reduce variance in predictions. Each tree was restricted to a maximum depth of 3 which helps to prevent overfitting. The minimum number of samples required to split an internal node was set to 5, and each leaf node contained at least 2 sample. At each split, the number of features considered was the square root of the total number of features, a common practice to enhance randomness and reduce correlation among trees.

### Analyses of models

Model performance was assessed using the Receiver Operating Characteristic (ROC) curve and the Area Under the Curve (AUC) as a measure of discrimination ability. Additionally, classification accuracy (correct classifications/total samples) was calculated to evaluate overall predictive performance. Metrics are reported as mean ± standard deviation across the 10 splits.

To identify which parts of the time-resolved signals contributed to the classification, we used the built-in impurity-based feature importance (mean decrease in Gini impurity) from scikit-learn. Feature importance scores were averaged across trees within each forest and then across the 10 splits and mapped back to the time axis to visualize which phases of the PORH protocol were most informative.

### Statistical test

Differences in baseline characteristics between low- and high-risk groups in the full cohort were assessed using the Mann–Whitney U-test for continuous variables and the $\chi^{2}$-test for categorical variables. Across the four subgroups (low-risk females, high-risk females, low-risk males, high-risk males), continuous variables were compared using the Kruskal–Wallis H-test. P-values from the four-group comparisons in Table 1 were adjusted for multiple testing using the Bonferroni correction.

Peak perfusion and peak oxygen saturation were compared across the four subgroups using the Kruskal–Wallis H-test. For these analyses, p-values were adjusted for multiple testing using the Benjamini–Hochberg procedure.

## Results

### Clinical characteristics

Of the 3,177 participants with valid microvascular measurements, 1,645 met the low-risk criteria and 761 met the high-risk criteria; the remaining participants fell between the two risk strata or had missing data on SCORE2 and were not included (Fig. 1). Clinical characteristics of the low- and high-risk groups are shown in Table 1. As expected, high-risk individuals were older, and more often current smokers, and had higher blood pressure, higher body mass index (BMI), higher non-HDL cholesterol and higher hsCRP than low-risk individuals.

**Table 1:** Clinical characteristics of the study population, stratified by SCORE2-based cardiovascular risk group for the full cohort and separately for females and males

Variable	All		*p*	Female		Male		*p*
	Low-risk (*n* = 1,645)	High-risk (*n* = 761)		Low-risk (*n* = 1,134)	High-risk (*n* = 181)	Low-risk (*n* = 511)	High- risk (*n* = 580)	
Age, years	55.7 ± 3.9	60.3 ± 3.8	< 0.001	56.4 ± 4.0^a^	59.9 ± 4.0^b^	54.0 ± 3.0^c^	60.4 ± 3.8^b^	< 0.001
Systolic blood pressure, mmHg	126 ± 15	144 ± 19	< 0.001	127 ± 16^a^	142 ± 22^b^	125 ± 11^a^	144 ± 18^bc^	< 0.001
Diastolic blood pressure, mmHg	81 ± 9	88 ± 11	< 0.001	81 ± 10^a^	88 ± 12^b^	79 ± 8^c^	88 ± 11^b^	< 0.001
Body mass index, kg/m^2^	26.1 ± 4.3	28.6 ± 4.5	< 0.001	26.1 ± 4.6^a^	28.7 ± 5.5^b^	26.0 ± 3.3^a^	28.6 ± 4.1^b^	< 0.001
Total cholesterol, mmol/L	5.5 ± 1.0	5.4 ± 1.3	0.028	5.7 ± 1.0^a^	5.4 ± 1.4^bc^	5.2 ± 0.9^c^	5.4 ± 1.3^bd^	< 0.001
Non-HDL cholesterol, mmol/L	3.7 ± 0.9	4.0 ± 1.3	< 0.001	3.7 ± 1.0^ab^	3.9 ± 1.4^ab^	3.6 ± 0.9^b^	4.1 ± 1.3^c^	< 0.001
hsCRP, mg/L	1.8 ± 3.8	2.3 ± 4.0	< 0.001	1.9 ± 4.4^a^	2.7 ± 3.5^b^	1.4 ± 2.0^c^	2.2 ± 4.2^d^	< 0.001
Current smoker	62 (3.8)	164 (21.6)	< 0.001	58 (5.1)^a^	54 (29.8)^b^	4 (0.8)^c^	110 (19.0)^d^	< 0.001
Diabetes mellitus	0	233 (30.6)	< 0.001	0^a^	84 (46.4)^b^	0^a^	149 (25.7)^c^	< 0.001
Hypertension	185 (11.2)	288 (37.8)	< 0.001	132 (17.3)^a^	77 (42.5)^b^	53 (10.4)^a^	211 (36.4)^b^	< 0.001
Dyslipidemia	66 (4.0)	176 (23.1)	< 0.001	43 (5.7)^a^	50 (27.6)^b^	23 (4.5)^a^	126 (21.7)^b^	< 0.001
eGFR, mL/min/1.73 m^2^	82 ± 12	82 ± 12	n.s	82 ± 12^a^	83 ± 13^ab^	84 ± 11^b^	82 ± 12^ab^	0.004
Previous ASCVD	0	100 (13.1)	< 0.001	0^a^	34 (18.8)^b^	0^a^	66 (11.4)^b^	< 0.001
SCORE2, %	3.0 ± 1.1	9.8 ± 2.2	< 0.001	2.7 ± 1.1^a^	8.8 ± 1.2 ^b^	3.8 ± 0.8^c^	9.9 ± 2.3 ^b^	< 0.001

ASCVD – atherosclerotic cardiovascular disease, eGFR – estimated glomerular filtration rate, HDL – high-density lipoprotein, hsCRP – high-sensitivity C-reactive protein, SCORE2 – Systematic Coronary Risk Evaluation 2,

Low-risk: SCORE2 < 5% without diabetes or previous ASCVD. High-risk: SCORE2 > 7.5%, or diabetes, or previous ASCVD, or severe chronic kidney disease (eGFR < 30 mL/min/1.73 m²). Continuous variables are presented as mean ± SD and compared between low- and high-risk groups using the Mann–Whitney U-test, and across the four subgroups (low-risk females, high-risk females, low-risk males and high-risk males) using the Kruskal–Wallis H-test. Categorical variables are presented as counts (percentages) and compared using the $\chi^{2}$-test. P-values from the four-group comparisons were adjusted for multiple testing using the Bonferroni correction, whereas p-values from two-group comparisons are unadjusted. Means in a row without a common superscript letter (a–d) differ significantly (*P* < 0.05) based on post-hoc pairwise comparisons of the four-group analysis

Among variables not directly used by SCORE2, the high-risk female group diabetes was present in 46% of high-risk females versus 26% of high-risk males (*p* < 0.05). hsCRP was also consistently higher in females than males within each risk stratum.

### Oxygen saturation, not perfusion, drives random forest classification of low- vs. high-risk individuals

**Fig. 2:**
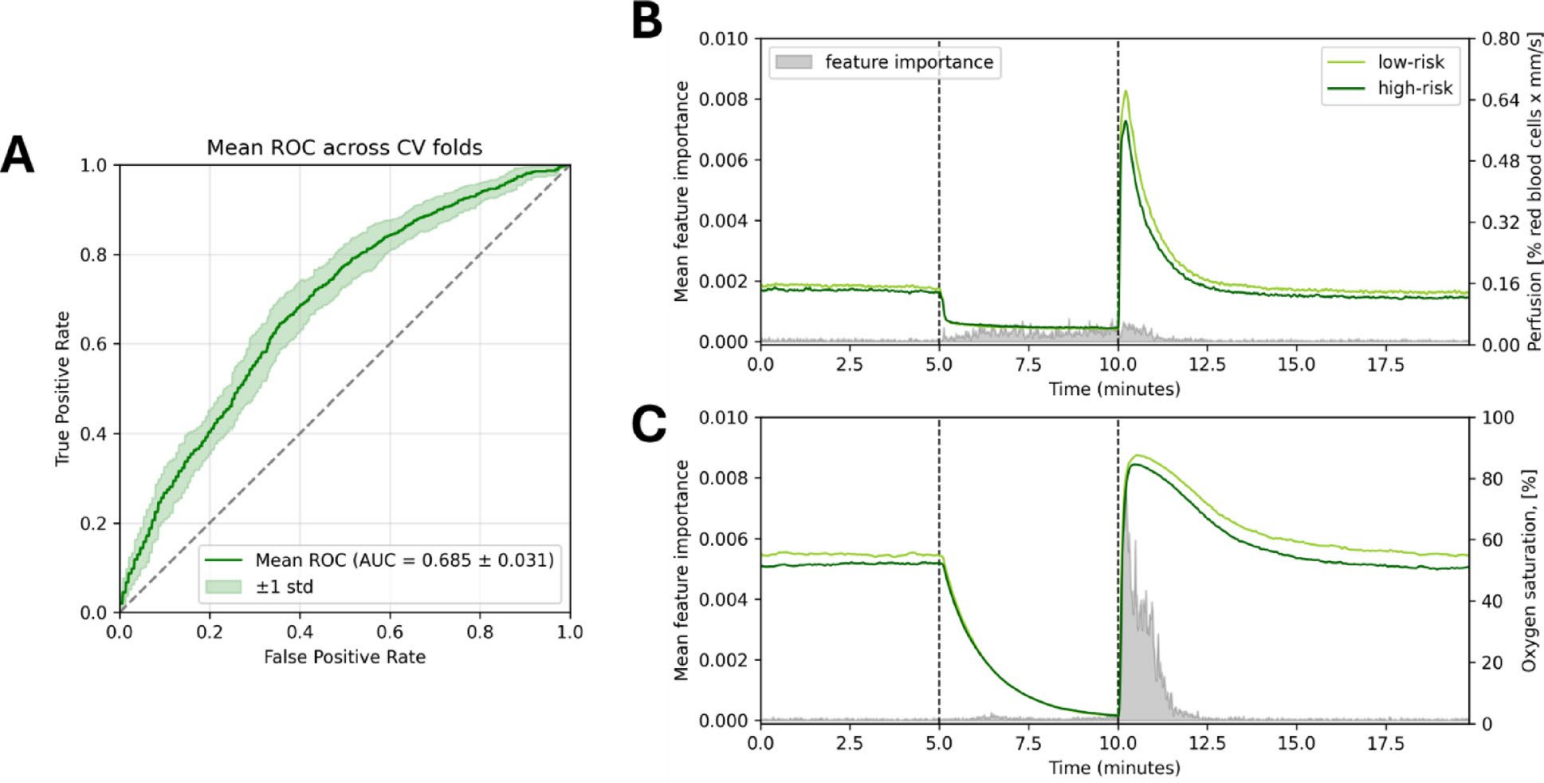
Random forest classification of low- vs. high-risk individuals (all participants). (**A**) Mean ROC across the 10 train/test splits. (**B**) Group-mean time-resolved microvascular perfusion in low-risk (light green) and high-risk (dark green) participants, with the shaded area showing the mean feature importance across the 10 models. (**C**) Group-mean time-resolved microvascular oxygen saturation for low- and high-risk participants, with feature importance overlaid as in B). Cuff inflation occurs at t = 5 min and release at 10 min (indicated with dashed lines)

We trained a random forest classifier on the combined time-resolved perfusion and oxygen saturation across all individuals (low-risk *n* = 1,645, high-risk *n* = 761) using 10 independent train/test splits. The combined model achieved a mean AUC of 0.685 (95% CI: 0.663–0.707) and accuracy of 0.642 (95% CI: 0.620–0.664) (Fig. 2A).

The group-mean microvascular parameters are shown in Fig. 2B. Both perfusion and oxygen saturation decreased at the onset of occlusion (t = 5 min) and showed a hyperemic overshoot after cuff release (t = 10 min). Both steady state values during pre- and post-occlusion as well as the magnitude of the hyperemic response values were on average higher in the low-risk group than in the high-risk group. The average feature importance across the 10 models (Fig. 2C) was concentrated in the post-occlusion hyperemic phase of the oxygen saturation, indicating that this part of the response drove classification.

To test which parameter contributed most, we trained separate models using oxygen saturation alone or perfusion alone. Oxygen saturation alone achieved similar performance to the combined model (AUC: 0.679, 95% CI: 0.654–0.704; accuracy: 0.624, 95% CI: 0.604–0.644)), while perfusion alone performed (AUC: 0.626, 95% CI: 0.601–0.651; accuracy: 0.592, 95% CI: 0.572–0.611), confirming that oxygen saturation was the more informative parameter in the full cohort.

### Peak perfusion contributes to risk discrimination in males but not in females

**Fig. 3:**
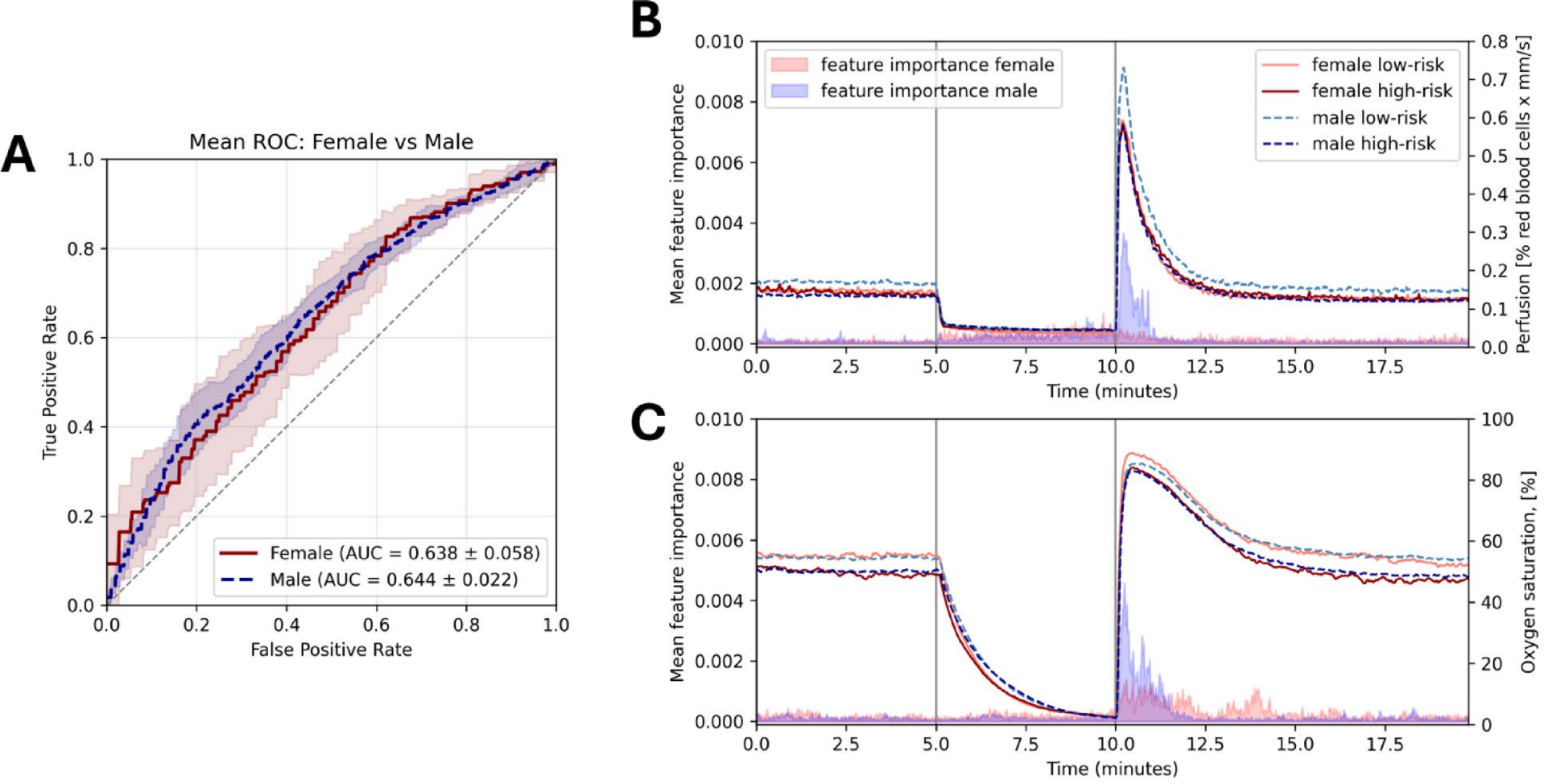
Sex-stratified random forest classification of low- vs. high-risk individuals (**A**) Mean ROC across the 10 train/test splits in female (red) and male (blue) models, with mean AUC. (**B**) Group-mean time-resolved microvascular perfusion for low-risk (light) and high-risk (dark) females (red) and males (blue), with the shaded area showing the mean feature importance across the models (**C**) Group-mean time-resolved microvascular oxygen saturation, with feature importance overlaid as in (B). Cuff inflation occurs at t = 5 min and release at 10 min (indicated with grey lines)

We trained random forest classifiers separately on the female and male subsets. Despite the smaller female dataset (*n* = 362), the female models achieved similar performance AUC 0.638 (95% CI: 0.596–0.679), accuracy 0.596 (95% CI: 0.563–0.629) to the male models (*n* = 1,022, AUC 0.644 (95% CI: 0.628–0.660), accuracy 0.601 (95% CI: 0.586–0.616)) (Fig. 3A). The group-mean time-resolved signals are shown in Fig. 3B (perfusion) and 3 C (oxygen saturation). Low-risk males exhibited a larger hyperemic increase in perfusion both in baseline and after cuff release than high-risk males, while no comparable difference was seen between low- and high-risk females. In females, the perfusion curves largely overlapped, particularly during the occlusion phase where the signal reflects a biological zero, with only limited separation after cuff release. Feature importance analysis reflected this pattern. Male models relied on both perfusion and oxygen saturation, whereas female models relied primarily on oxygen saturation. The informative regions also appeared to span different parts of the post-occlusion phase in males and females (Fig. 3B, C).

Sensitivity analyses with each signal alone supported this conclusion. Oxygen saturation alone achieved similar performance to the combined model in both sexes (females: AUC 0.631 (95% CI: 0.594–0.668), accuracy 0.608 (95% CI: 0.574–0.642); males: AUC 0.624 (95% CI: 0.607–0.641), accuracy 0.586 (95% CI: 0.567–0.605)) while perfusion alone performed worse in both sexes (females: AUC 0.587 (95% CI: 0.533–0.641), accuracy 0.573 (95% CI: 0.522–0.623); males: AUC 0.611 (95% CI: 0.589–0.633), accuracy 0.578 (95% CI: 0.564–0.592)). Oxygen saturation was therefore necessary for classification in both sexes while perfusion contributed additional information only in males.

**Fig. 4:**
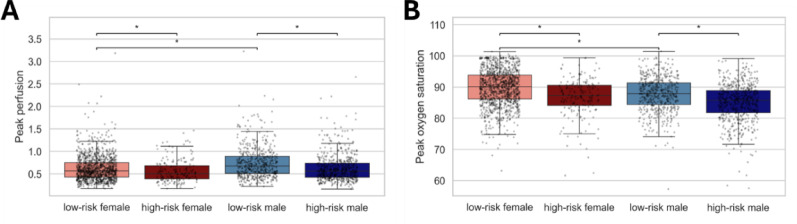
Peak perfusion and peak oxygen saturation. (**A**) Peak perfusion and (**B**) Peak oxygen saturation for the four subgroups. Boxplots show median (horizontal line), interquartile range, IQR, (box), and 1.5xIQR (whiskers). Individual observations are overlaid as jittered points. Brackets with an asterisk indicate Benjamini-Hochberg adjusted p-values < 0.05

To quantify the sex-specific patterns visible in the time-resolved signals, we compared peak perfusion (Fig. 4A) and peak oxygen saturation (Fig. 4B) across the four subgroups (*n* = 2,406). Low-risk individuals had higher peaks of both perfusion and oxygen saturation than high-risk individuals within each sex. Low-risk females had higher peak oxygen saturation than low-risk males, while low-risk males had higher peak perfusion than low-risk females (all comparisons *p* < 0.05, Benjamini-Hochberg adjusted).

## Discussion

In our study, we showed that time-resolved microcirculatory oxygen saturation and perfusion parameters carry different predictive information for CVD risk discrimination, and that the informative signal differs between males and females. In the combined cohort, oxygen saturation in the post-occlusive phase was the dominant signal driving classification, while perfusion contributed little. Importantly, these differences reflect not only classification performance but also underlying physiological variation. High-risk individuals exhibited an attenuated post-occlusion hyperemic response in oxygen saturation, suggesting impaired microvascular function. When models were trained separately by sex, oxygen saturation remained informative in both sexes, but perfusion provided additional information only in males. In males, reduced perfusion responses complemented the oxygen saturation signal, while in females, perfusion differences were less pronounced and showed limited discriminatory value.

Previous studies of sex differences in microvascular reactivity have produced inconsistent results. Some have reported greater vasodilatory reactivity in females than in males using LDF during PORH [18] or local heating [19], while others have found no sex differences during PORH assessed by nailfold capillaroscopy or transcutaneous oxygen partial pressure of the forearm [20]. In a previous analysis of a subgroup of 1765 individuals also included in this cohort, we reported higher peak oxygen saturation and lower peak perfusion in females than in males as part of a normative characterization of the cohort [9]. The present study extends this observation by showing that the same sex difference is reflected in the time-resolved signals used by the classifier, and that the two signals carry different weight for risk discrimination in males versus females.

There are several mechanisms that have previously been shown to be involved in sex-specific effects on microvascular and endothelial function and that may underlie the differences in weighting of perfusion versus oxygen saturation observed in this study. Vasodilatory responses involve multiple pathways including sensory nerves, nitric oxide (NO) dependence, prostanoid and adrenergic responsiveness, and the relative contribution of these pathways differs between sexes. Sensory nerve contribution to vasodilation is modulated by sex and by menstrual phase, as shown by local anesthetic blockade (EMLA) [19], and autonomic/sympathetic control of cutaneous vasoconstriction also shows sex differences [21]. Estrogen has both rapid non-genomic effects (activating endothelial nitric oxide synthase to increase NO production and subsequent vasodilation [22] and slower effects on vascular smooth muscle proliferation, re-endothelialization, endothelial inflammation, and reactive oxygen species [23]. These pathways may differentially influence the perfusion and oxygen saturation components of the PORH response, contributing to the differences observed in our analysis.

Whether the sex differences in peak perfusion and peak oxygen saturation reflect innate biological differences or differences in disease burden is difficult to disentangle from cross-sectional data. We have previously shown that peak oxygen saturation is associated with cardiovascular risk factors and with atherosclerosis [10, 11]. SCORE2 is based on sex-specific prediction models that account for differences in the underlying incidence of cardiovascular events [7]. As a result, the same levels of conventional risk factors generally yield a lower absolute 10-year risk in females than in males. Consequently, females often require a higher cumulative risk factor burden or older age to reach equivalent risk thresholds, as in line of our results where the high-risk female group had a substantially heavier burden of clinically established disease than the high-risk male group, including more than twice the prevalence of diabetes (46% versus 26%). This consequence, where females require a higher cumulative risk factor burden to reach equivalent risk thresholds in SCORE2, reflects calibration to population-level data rather than a lower etiological importance of risk factors in females, which is essential to consider when interpreting risk. In fact, there are data showing that certain risk factors (such as diabetes and smoking) may be associated with a greater relative increase in risk in females [24, 25]. Sex-specific patterns in the microvascular signals may therefore reflect a combination of innate physiology, disease burden, and treatment effects, and these factors cannot be separated in the present analysis.

Our random forest classifier achieved modest predictive performance (AUC 0.63–0.67) when distinguishing low- from high-risk individuals using only time-resolved microvascular signals (oxygen saturation and perfusion). These AUC values are lower than those reported in proteomics-based studies of CVD prediction (AUC 0.70–0.94) [26–29] and substantially lower than a study by Yan et al. that achieved 88–96% accuracy for diagnosing hypertension and coronary heart disease using peripheral blood flow data [30]. However, direct comparison is difficult: proteomics measure blood biomarkers, while Yan et al. [30] used physiologically engineered features (vascular resistance, compliance, flow inertia) rather than raw time-series signals. In a recent study by Maldonado-Garcia et al. [31], retinal optical coherence tomography (OCT) imaging of the microcirculation was used in a random forest classifier to predict cardiovascular events within five years. They achieved an AUC of 0.75 when combining OCT features with clinical data and identified microvasculature as the most informative feature. Like our study, the prediction was based on a non-invasive measure of the microcirculation. In contrast to our work, their model combined retinal structural imaging with clinical variables to predict events rather than time-resolved data to discriminate risk profiles. Our use of raw signals trades predictive accuracy for the ability to identify which time-resolved features the model relies on, which was the primary aim of the present analysis.

Several methodological limitations should be considered. The random forest is data-driven and identifies which signal regions are informative for classification but does not provide a mechanistic explanation. By design we excluded participants with SCORE2 between 5% and 7.5% without additional risk-defining conditions to obtain two clearly separated risk strata, and this group represents approximately one quarter of the sub-study cohort and may differ from both included groups. The female and male subsets were highly imbalanced before class balancing (1,134 vs. 181 in females; 511 vs. 580 in males), and the small number of high-risk females in particular may limit the generalizability of the female-specific findings. In addition, the use of a modified SCORE2 threshold (7.5% instead of the conventional 10%) may limit comparability with standard risk categories and affect the generalizability of the findings. The all-individuals analysis is dominated by the larger female low-risk group, which may explain why perfusion appears uninformative in the combined cohort despite contributing in the male-only analysis. Finally, feature importance of random forests may overemphasize correlated predictors which is a concern with autocorrelated time-series; permutation importance or alternative model classes would be useful for sensitivity analyses in future work.

## Conclusions

In summary, time-resolved microvascular oxygen saturation and perfusion signals carry information for CVD risk discrimination. Oxygen saturation in the post-occlusive hyperemic phase contributes to risk classification in both sexes, while perfusion contributes only in males. Further research is needed to identify the physiological mechanisms underlying the differing importance of perfusion and oxygen saturation, and to determine whether these time-resolved features add prognostic value beyond established risk scores.

## Data Availability

Data are available from the authors upon reasonable request by contacting the corresponding author and study organization (www.scapis.org). Code used for analysis is available at GitLab: https://gitlab.liu.se/ISBgroup/projects/cvd_risk_micro.
